# The dual role of empagliflozin: Cardio renal protection in T2DM patients

**DOI:** 10.1016/j.amsu.2022.104555

**Published:** 2022-09-02

**Authors:** Aimen Shafiq, Eman Mahboob, Muhammad Ammar Samad, Mohammad Hassam Ur Rehman, Zoaib Habib Tharwani

**Affiliations:** Faculty of Medicine, Dow Medical College, Dow University of Health Sciences, Karachi, Pakistan

**Keywords:** Empagliflozin, Type 2 diabetes mellitus, Cardio protective, Reno protective, Side effects

## Abstract

Empagliflozin (Jardiance®) is an insulin independent antihyperglycemic agent used in treatment of T2D.The drug is a sodium glucose cotransporter-2 (SGLT2) inhibitor approved in USA and Europe and other countries of the world. As empagliflozin demonstrates cardioprotective and Reno protective properties its prime target are patients having CVD and CKD complicated by T2D.

This review sheds light on mechanism of action of the drug and with the help of clinical outcomes establishes the use of empagliflozin in T2D patients.

Although empagliflozin is a well-tolerated and easy to administer drug, it has some side effects and contraindications which are discussed in the article to help the reader weigh its beneficial effects against its adverse effect and understand its use in clinical medicine.

## 1 Introduction

The highest percentage of morbidity and mortality in Diabetes type 2 patients is due to cardiovascular diseases (CVD) [1]. Addressing high blood glucose level and other risk factors for CVD, such as high blood pressure, obesity, and dyslipidaemia by modifying lifestyles and use of pharmacological therapies are the mainstay treatment for improving fatality in Diabetes type 2 patients [[2], [3]].

Antidiabetic agents (ADA) that inhibit Sodium glucose cotransporter-2 (SGLT2) such as Gliflozins are used as pharmacological intervention in diabetic patients [2,4]. Gliflozins are unique ADAs as they are insulin independent agents which lowers the serum glucose level by increasing the excretion of glucose in urine (Glucosuria). Along with decreasing glucose serum levels these drugs also target the metabolic and hemodynamic anomalies that increase the risk for CVDs [5,6].

Originally known as compound BI 10773, Empagliflozin is a U.S. Food and Drug Administration (FDA) approved Gliflozin which was Engineered by Boehringer Ingelheim Pharmaceuticals (Germany) [7]. Empagliflozin have an empirical formula of C23H27ClO7 and molecular mass of 450.91 g/mol [8] had a better anti diabetic effect than dapagliflozin (another gliflozin) in lowering HbA_1c_ and improving cardiovascular symptoms [9].

In patients with T2D and CVDs empagliflozin proved to be protective against cardiac and renal effects independently of the glucose control making it an approved drug in the USA [10] and the Europe [11].

This review emphasizes and describes the mechanism of action, clinical outcomes, side effects and contraindications in the treatment of patients with Type 2 Diabetes along with a history of CVD.

### 1.1 Mechanisms of cardiovascular protection

The mechanism by which empagliflozin- An SGLT2 inhibitor provides cardiovascular (CV) protection particularly in heart failure is not completely understood considering the absence of SGLT2 expression in cardiac tissue [[12], [13]]. There have been several theories that empagliflozin produces its CV effects by inhibiting the sodium-hydrogen exchanger 1 (NHE1) in the heart muscles and sodium-hydrogen exchanger 3 (NHE3) located in the proximal tubule which is accountable for most of the electrolyte and water reabsorption in the kidneys, thus reducing the preload via diuresis and natriuresis [14].

The EMPA-REG OUTCOME trail [15] and cardiovascular outcome trials with DPP4 inhibitors suggest that there was only a minor change in HbA_1c_ levels in patients receiving empagliflozin as compared those who did not receive the drug [16,17]. Further the trials also suggest that the reduced atherosclerotic events cannot be considered as CV benefits of empagliflozin [15].

**Fig. 1:**
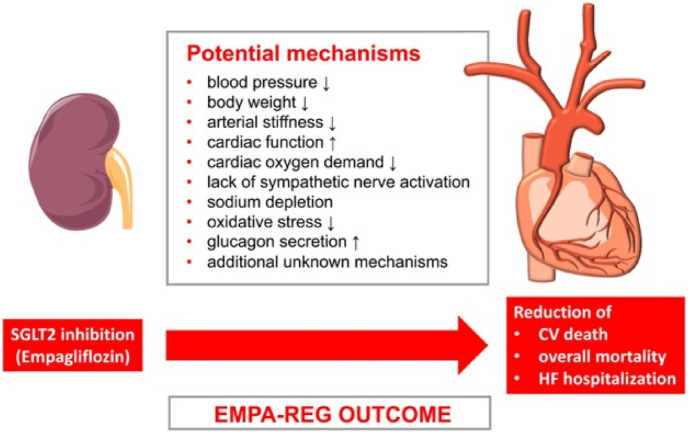
Cardiovascular protective mechanisms by Empagliflozin.

There are many mechanisms which are not related to atherosclerosis and provide cardiovascular benefits with empagliflozin (Fig. 1). [13,18]. Firstly, empagliflozin signifanctly reduces Systolic Blood Pressure (SBP) and arterial stiffness [19] that leads to better oxygen consumption by myocardium and hence lowers the cardiac afterload [2,13]. It also helps by reducing the body weight slightly [12]. Secondly empagliflozin also contributes by lowering the plasma volume by diuresis i.e., the increased excretion of sodium and glucose in urine. The SGLT2 inhibition has a direct effect on the myocardium as it leads to excessive sodium excretion in urine, rapidly decreasing the total-body content of sodium. Also, the increased NACL delivery to the macula densa cells located in the distal convoluted tubule of the kidneys leads to the inhibition of Renin angiotensin system (RAAS) [12]. Empagliflozin causes the shifting of fuel metabolism from fat oxidation to ketone bodies in myocardium and kidney as a result there is an improve in oxygen consumption in mitochondria which contributes to the restoration of heart and kidneys [20]. Also the reduced uptake of carbohydrates by the tissues, low plasma insulin and high plasma glucagon due to chronic glucosuria, increases lipolysis and fat consumption in the body [20]. Other effects of CV benefits with empagliflozin have been observed in animal models which include anti oxidative, inflammatory and apoptotic effects of SGLT2 inhibition [12]. We need further studies to provide a clear picture for the mechanisms of CV benefits.

### 1.2 Mechanisms of renal protection

In the proximal convoluted tubules (PCT) of the kidney, SGLT2 are present where maximal glucose reabsorption occurs in the blood. Empagliflozin inhibits these transporters int the PCT of the kidneys and lead to glucosuria hence lowering the blood glucose levels in patients with T2DM [21].

**Fig. 2:**
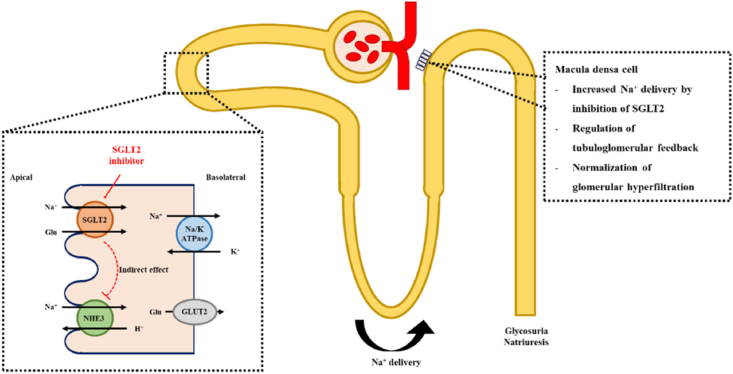
Mechanism of SGLT2 inhibition and the regulation of tubuloglomerular feedback by Empagliflozin.

The molecular as well as cellular mechanisms for CVD and chronic kidney disease (CKD) in patients of type 2 diabetes mellitus (T2DM) have been found to be similar [22]. Hence the mechanisms in the EMPA-REG OUTCOME trial which cause CV benefits can also be the reasons for improved renal outcomes. Like when there is increased luminal sodium chloride concentration, the macula densa cells in the distal convoluted tubule of the kidney detect these changes and leads to the constriction of afferent renal arterioles, lowers the glomerular filtration rate (GFR) and normalizes the intraglomerular pressure via tubuloglomerular feedback (Fig. 2) [23,24]. Furthermore, a reduction of approximately 6–8 mm Hg pressure in glomerular hypertension and type 1 diabetes has been seen with empagliflozin even though there is a low-pressure environment in the renal glomeruli [25].

There were five different trails performed in patients of T2DM and albuminuria [[26], [27], [28], [29], [30]]. according to these trials empagliflozin benefits by reducing the urinary albumin-to-creatinine ratio (ACR) by a much greater extent [24]. It was also proved that this decrease of ACR was not due to the reduced HbA_1c,_ body weight or SBP. Furthermore, the small decreases in eGFR due to SGLT2 inhibition in the starting three to four weeks of treatment with the drug suggests that the reduced intraglomerular pressure also contributes to a decrease in ACR [24].

Empagliflozin also produces a favorable effect on the systemic and neurohormonal pathways like RAAS, and markers of arterial stiffness and vascular resistance including pulse pressure, ambulatory arterial stiffness, mean arterial pressure and double product, and lastly on the serum uric acid levels and hence majorly reduces the risk for renal dysfunctions in patients with T2DM [23,24]. Further studies are needed to prove if empagliflozin plays a role in altering serum creatinine and other renal functions by affecting the plasma volume or renal perfusion [23,24].

## 2 Clinical outcomes of empagliflozin

### 2.1 Cardiac efficacy of empagliflozin- the EMPA-REG trial

A randomized, multicenter, double-blind, placebo-controlled trial of empagliflozin in people with T2D and established CVD was called the EMPA-REG OUTCOME study. 7028 participants from 42 different nations were randomly assigned to receive one of two dosages of the medication empagliflozin (10 or 25 mg) or a placebo in addition to receiving the best care possible for their T2D and CV risk factors. Based on the combined results of the empagliflozin 10 mg or 25 mg groups vs placebo, the primary statistical analysis was performed. The mean follow-up time was 3.1 years [31].

#### 2.1.1 Cardiovascular outcomes

Empagliflozin (10.5%; 490/4687) improved the primary outcomes (CV mortality, non-fatal MI, and non-fatal stroke) compared to placebo (12.1%; 282/2333); HR, 0.86. (95% CI: 0.74, 0.99). The primary outcome was improved by a 38% drop in CV mortality, but there was no detectable decline in MI or stroke. Empagliflozin reduced CV mortality incredibly quickly (with just under 3 months of therapy), and the expanding gap between the treatment and control survival curves suggests a persistent treatment advantage. Additionally, empagliflozin decreased mortality from all causes by 32%. The decrease in CV deaths was seen in a variety of subgroups, including those with preexisting CVD, age, gender, rising or falling HbA1c levels, and changes in HbA1C and kidney function [[31], [32], [33]].

#### 2.1.2 Heart failure outcomes

The evaluated result of HF hospitalization was lowered by empagliflozin by 35%. Empagliflozin significantly decreased deaths from HF and other non-adjudicated markers of HF outcomes, such as investigator-reported HF and the use of loop diuretics [32]. With substantial advantages throughout subgroups including race, age, estimated glomerular filtration rate, CV medications, usage of glucose-lowering drugs (including insulin), and diuretics, the incidence of HF hospitalization or Cardiovascular mortality was reduced for empagliflozin (5.7%) compared to placebo (8.5%). Fewer patients receiving empagliflozin (13.5%) than those getting a placebo died from CV causes among those who were hospitalized for HF (24.2%). The 10% of patients who had a prior history of HF showed comparable decreases in CV deaths and HF outcomes to the whole group. Patients with HF at all levels of risk showed empagliflozin's benefits for their CV [34]. The decline in CV mortality and HF hospitalization for empagliflozin versus placebo was constant throughout the risk subgroups in the general population using the Clinical Health Aging and Body Composition HF risk score, with an HR (95% CI) of 0.71 (0.52, 0.96) in the low - risk group, 0.52 (0.36, 0.75) in the high-risk group, and 0.55 (0.30, 1.00) in the very high-risk group. A sum of 958 individuals (13.6%) had a heavy HF load (HF reported by the investigator, HF hospitalization, or HF at baseline as an adverse event). Patients with an HF load had a nearly fourfold higher rate of cardiovascular death, accounting for 38% of all CV fatalities in the EMPA-REG OUTCOME trial. Patients without an HF burden experienced a significant mortality benefit from empagliflozin (2.7% versus 4.2%, respectively) despite the fact that the absolute benefit was higher (4.9%) in the population with an HF burden (10.4% versus 15.3%, respectively).

### 2.2 Clinical efficacy of empagliflozin in type 2 diabetes mellitus

#### 2.2.1 Glucose lowering effect

In individuals with T2DM, treatment with empagliflozin reduces HbA1c by 0.79% with monotherapy and 0.61% with add-on treatments to other glucose-lowering medications, according to a meta-analysis of 45 randomized trials [35]. Empagliflozin enhances time-in-range (TIR, the proportion of time spent in the target glucose range between 70 and 180 mg/dL) as measured by continuous glucose monitoring and minimizes postprandial and fasting hyperglycemia [36,37].

#### 2.2.2 Body weight, blood pressure, and other metabolic parameters

Empagliflozin medication decreases body weight by 1.7 kg (2.4%) in T2DM patients, as well as diastolic and systolic blood pressure by 2 and 4 mmHg, respectively, without raising the heart rate [35]. Treatment with empagliflozin has also been shown to decrease serum uric acid by 0.3–0.9 mg/dL, serum triacylglycerol by 1–9%, and raise blood HDL cholesterol by 6–9% in T2DM patients [35].

#### 2.2.3 Hypoglycemia

Empagliflozin has a lower risk of hypoglycemia, and when administered as monotherapy, this risk is equivalent to that of a placebo [35,38]. However, using empagliflozin along with insulin and/or insulin secretagogues may raise the risk of hypoglycemia. Hence, in order to prevent hypoglycemia, the dosage of insulin and/or insulin secretagogues have to be lowered when used in conjunction with empagliflozin. To prevent the onset of diabetic ketoacidosis, it is advised to adjust the insulin dosage between 10 and 20% of the overall dose [39].

#### 2.2.4 Beta cell function

Rather than increasing insulin production, empagliflozin improves beta-cell function [40,41] by reducing glucotoxicity and perhaps even beta-cell burden [[42], [43], [44]] since they decrease plasma glucose concentration independently of insulin. After taking empagliflozin, glucagon secretion rises, probably as a result of the body losing glucose quickly [45,46]. Increased plasma glucagon concentrations also aid in increasing lipolysis and lowering visceral adiposity and liver fat [47]. Although there are mixed results [48,49] and more investigation is necessary, it has also been suggested that empagliflozin may have a direct impact on alpha cells [50,51].

#### 2.2.5 Renal outcomes of the EMPA-REG trial

The sodium-glucose cotransporter 2 (SGLT2) inhibitor, empagliflozin, was the subject of the Empagliflozin Cardiovascular Outcome Event Trial in Type 2 Diabetes Mellitus Patients-Removing Excess Glucose (EMPA-REG OUTCOME) [31], the very first clinical trial to show supremacy for the primary endpoint. The trial also identified a particularly significant decrease in the risk of cardiovascular death in T2DM patients. Though the trial was originally conducted to demonstrate the effects of empagliflozin on cardiac events, its renoprotective role suggested a new chance of therapy for patients with T2DM.

The cumulative kidney outcome in the EMPA-REG OUTCOME study was the prevalence of occurrence or deteriorating nephropathy, which was characterized as the development of macroalbuminuria, a twofold increase of creatinine concentration, with eGFR of 45 ml/min/1.73 m2, starting of dialysis, or mortality from kidney impairment [33]. Compared to placebo, empagliflozin (12.7%) improved new or worsening nephropathy (18.8%). The three post-hoc kidney cumulative results were starting kidney replacement therapy, a twofold rise in plasma creatinine, or mortality from renal illness. Compared to placebo, empagliflozin had a 46% lower risk of these events [33]. The likelihood of developing macroalbuminuria was decreased by empagliflozin by 38%, the chance of increasing creatinine level twofold was decreased by 44%, and the incidence of beginning renal replacement therapy was increased by 55%. 2250 (67%) of the 7020 patients overall had pervasive nephropathy at baseline, which was considered as an eGFR of less than 60 mL/min/1.73 m2 and/or urinary albumin to creatine ratio (UACR) of more than 300 mg/g. Empagliflozin decreased HF hospitalization risk by 39%, CV death risk by 29%, all-cause mortality risk by 24%, and all-cause hospitalization risk by 19% in individuals with recurrent renal dysfunction.

In accordance with the baseline UACR score (normoalbuminuric: UACR 30 mg/g; microalbuminuria: UACR 30–300 mg/g; and macroalbuminuria: UACR >300 mg/g), pre-determined and post-hoc studies were carried out to evaluate alterations in UACR scores. 59% of patients were having normoalbuminuric at baseline, followed by microalbuminuria (29%) and macroalbuminuria (11%) [33,52]. As compared to placebo, with the use of empagliflozin, there was a significant drop in UACR in individuals with micro- or macro-albuminuria that persisted at weeks 12 and 164 [53].

## 3 Safety and adverse effects

**Fig. 3:**
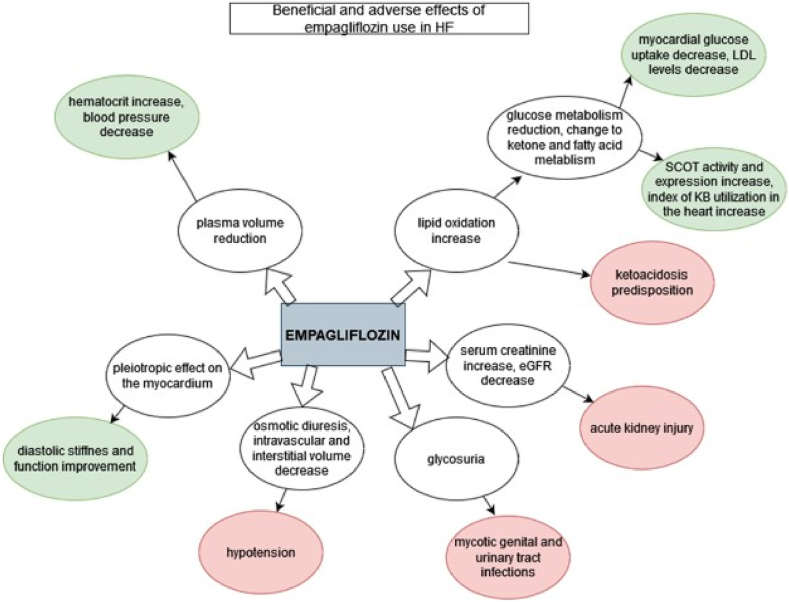
Beneficial and adverse effects of empagliflozin use in HF. [Green: beneficial effects; red: adverse effects]. (For interpretation of the references to colour in this figure legend, the reader is referred to the Web version of this article.)

Empagliflozin provides many benefits but consideration should be given to the potential dangers and drawbacks of taking SGLT-2 inhibitors in addition to potential advantages when determining the clinical usefulness of empagliflozin (Fig. 3). If the Glomerular Filtration Rate (GFR) is less than 20 mL/min/1.73 m2 then empagliflozin use is not advised [54]. Empagliflozin dosage restrictions may frequently be based on GFR criteria in HF patients because these patients frequently have a concomitant renal illness [55]. Additionally, empagliflozin is not prescribed to people with type 1 diabetes who also have heart failure or diabetic ketoacidosis [56,64]. Moreover, use in pregnant women in their second and third trimesters and anyone who has experienced a significant allergic response to empagliflozin is contraindicated [56].

Ketoacidosis, acute kidney injury, dyslipidemia, hypoglycemia in patients taking insulin, hypotension (especially in patients taking diuretics, ARBs, or ACE-inhibitors), genital mycotic infections, hypotension (especially in patients taking ACE-inhibitors, ARBs, or diuretics), and, very rarely, peripheral amputations and Fournier gangrene [63] are some of the side effects of empagliflozin [54,56]. The side effects associated with empagliflozin are consistent with the SGLT-2 class's unique kidney mode of action [57]. Empagliflozin can result in symptomatic hypotension because it induces osmotic diuresis and lowers interstitial and intravascular volume, specifically in elderly people, those using hypotensive medications, people with low systolic blood pressure, and people with kidney insufficiency [54,[56], [57]]. A dose adjustment is necessary because loop diuretics, which are frequently prescribed to HF patients, could potentially react with SGLT2-inhibitors [58]. Patients with symptomatic hypovolemia or more serious side effects, like ketoacidosis, may need to stop taking empagliflozin. For patients with clinically diagnosed hypovolemia or with ketoacidosis, empagliflozin discontinuation is indicated [58].

Euglycemic ketoacidosis has been recorded more frequently while taking SGLT-2 inhibitors, which necessitates ketoacidosis avoidance by switching from oral medication to IV insulin. Hospitalization for any emergency health condition or surgery predisposes individuals to this disease [54]. Empagliflozin induces volume depletion [60], lowers eGFR, and raises blood creatinine levels, all of which increase the risk of ketoacidosis and may precipitate acute kidney injury. Kidney function must be initially assessed and routinely checked when using SGLT-2 inhibitors [56]. In the setting of SGLT2-inhibitor therapy, urinary tract infections and mycotic vaginal infections are frequent [59], but they can be avoided by practicing strict hygiene [56].

A recent FDA caution was just released highlighting the heightened risk of bone fractures driven by reduced bone mineral density and osteoporosis with the use of SGLT2 inhibitors [61]. It is suspected that SGLT2 inhibitors raise serum phosphate levels, which may have a negative impact on bone metabolism [62]. Although empagliflozin has not related to the negative effects on bone resorption, it is important to take into account the risks of osteoporosis associated with its use as well as the impact on patients with heart failure having weaker bones.

## 4 Conclusion and future directions

The most recent studies on the benefits and drawbacks of empagliflozin, a drug used to treat both heart failure and diabetes mellitus, have been emphasized in this review. Empagliflozin is the first drug to demonstrate advantages in cardiovascular events in the setting of devoted CV outcomes studies and is a paradigm-shifting treatment for T2DM therapy. Across a number of patient populations, including those with or without HF at baseline, there is a persistent, significant efficacy in lowering HF hospitalizations and cardiovascular deaths. These advantages could be achieved through empagliflozin's influence on uric acid, body weight, oxidative stress, arterial stiffness, blood pressure, and fat distribution. In contrast to other T2DM therapy, empagliflozin has a distinct effect on natriuresis and diuresis. People with HF frequently have complicated medical histories with several illnesses, most frequently diabetes and kidney failure. A medication like empagliflozin with such broad pleiotropic effects that has the capability to delay the point of crossover between these conditions appears particularly useful because several of these comorbidities can both be a source and a consequence of heart failure.

However, the risks associated with empagliflozin use should be addressed when deciding whether to include it in treatment. Significant renal dysfunction, the second and third trimesters of gestation, and type 1 diabetes are the major contraindications. In HF patients receiving diuretics and ACE inhibitors, the potential for experiencing hypotension should also be considered, particularly in those with low blood pressure at the start of their treatment. The patients must be made aware of the potential genitourinary infection and adverse effects of empagliflozin and given guidance on how to avert them to avoid non-compliance of the medication. Additionally, it is uncertain whether the advantages of empagliflozin are specific to the medication or if there is a class effect. As a result, more extensive clinical trials are needed to verify the efficacy of other SGLT2 inhibitors in the management of HF and T2DM as well as the benefits and drawbacks of their use concerning the contraindications and negative effects they encourage. Furthermore, to investigate the impact of empagliflozin on hemodynamics, left ventricular function and structure, New York Heart Association class is to decide whether there are differences between HF with reduced or preserved ejection fraction, additional research in specialized HF populations with pre-specified HF analyses is required.

Future trials of empagliflozin could be able to better understand how it affects markers of the severity of HF and how well it responds to treatment by using measurements of cardiac function and biomarkers like natriuretic peptides. The sustainability of the HF and cardiovascular and renal advantages as well as the possibility of late-onset unfavorable effects require long-term studies.

## Ethical approval

N/A.

## Sources of funding

None.

## Author contribution

All authors equally contributed.

## Trial register number

1. Name of the registry: N/A
2. Unique Identifying number or registration ID: N/A
3. Hyperlink to your specific registration (must be publicly accessible and will be checked): N/A

## Guarantor

N/A.

## Consent

N/A.

## Provenance and peer review

Not commissioned, externally peer reviewed.

## Declaration of competing interest

None declared.
